# Breast cancer associated a2 isoform vacuolar ATPase immunomodulates neutrophils: potential role in tumor progression

**DOI:** 10.18632/oncotarget.5439

**Published:** 2015-10-09

**Authors:** Safaa A. Ibrahim, Gajendra K. Katara, Arpita Kulshrestha, Mukesh K. Jaiswal, Magdy A. Amin, Kenneth D. Beaman

**Affiliations:** ^1^ Department of Microbiology and Immunology, Rosalind Franklin University of Medicine and Science, North Chicago, IL, USA; ^2^ Department of Microbiology and Immunology, Faculty of Pharmacy, Cairo University, Giza, Egypt

**Keywords:** breast cancer, a2 vacuolar ATPase, tumor associated neutrophils, angiogenesis, invasion

## Abstract

In invasive breast cancer, tumor associated neutrophils (TAN) represent a significant portion of the tumor mass and are associated with increased angiogenesis and metastasis. Identifying the regulatory factors that control TAN behavior will help in developing ideal immunotherapies. Vacuolar ATPases (V-ATPases), multi-subunit proton pumps, are highly expressed in metastatic breast cancer cells. A cleaved peptide from a2 isoform V-ATPase (a2NTD) has immunomodulatory role in tumor microenvironment. Here, we report for the first time the role of V-ATPase in neutrophils modulation. In invasive breast cancer cells, a2NTD was detected and a2V was highly expressed on the surface. Immunohistochemical analysis of invasive breast cancer tissues revealed that increased neutrophil recruitment and blood vessel density correlated with increased a2NTD levels. In order to determine the direct regulatory role of a2NTD on neutrophils, recombinant a2NTD was used for the treatment of neutrophils isolated from the peripheral blood of healthy volunteers. Neutrophils treated with a2NTD (a2Neuɸ) showed increased secretion of IL-1RA, IL-10, CCL-2 and IL-6 that are important mediators in cancer related inflammation. Moreover, a2Neuɸ exhibited an increased production of protumorigenic factors including IL-8, matrix metaloprotinase-9 and vascular endothelial growth factor. Further, functional characterization of a2Neuɸ revealed that a2Neuɸ derived products induce *in vitro* angiogenesis as well as increase the invasiveness of breast cancer cells. This study establishes the modulatory effect of breast cancer associated a2V on neutrophils, by the action of a2NTD, which has a positive impact on tumor progression, supporting that a2V can be a potential selective target for breast cancer therapy.

## INTRODUCTION

The interaction between tumor cells and inflammatory cells has important role in cancer establishment and progression; however, this interaction has not been well defined. Tumor-associated macrophages (TAM) and tumor-associated neutrophils (TAN) exist in almost all solid neoplasms and can control cancer growth [1]. TAN infiltration and poor clinical outcomes are positively correlated in many tumors [2]. TAN represent a significant portion of the total mass of invasive breast carcinomas and are proposed to be an important component involved in breast cancer chemoresistance and metastasis [3–5]. Tumors secrete factors into the tumor microenvironment that induce a wound repair response from both TAM and TAN [2, 6, 7]. In an *in vitro* co-culture breast cancer model, GM-CSF released from breast cancer cells stimulates oncostatin-M secretion from neutrophils leading to an increase of their invasive capacity [3]; however, factors regulating TAN behavior in breast cancer remain unclear.

Neutrophils are important members of the innate immune system and represent a first line defense against infections. In murine models of lung cancer, TGF-β drives TAN to acquire an N2 or protumoral phenotype [7]. TAN are proposed as key mediators of tumor progression and produce several factors that promote angiogenesis, invasion and metastasis, stimulating tumor growth and modulating the antitumor immunity [2]. TAN can secrete various immunoregulatory cytokines and chemokines; proinflammatory mediators IL-8, IL-6, TNF-α, IL-1α, IL-1β, CCL2, CXCL-1, CXCL-2 and CCL-17, anti-inflammatory cytokine IL-1RA, as well as Arg-1 [8, 9]. In addition, TAN secrete angiogenic factors such as IL-8 and VEGF and are considered to be a primary source of matrix-metalloproteinases-9 (MMP-9) in human hepatocellular carcinoma [9, 10].

Tumor associated vacuolar ATPases (V-ATPases) are multi-subunit proton pumps, expressed on the plasma membrane of the cells as well as on the intracellular organelles membrane. V-ATPases help maintain the acidic tumor microenvironment thereby supporting tumor growth [11]. A number of recent studies elaborate the role of V-ATPases in supporting breast cancer growth and progression [12–15]. Our previous studies have shown that the a2 isoform of ‘a’ subunit of V-ATPase (a2V) plays a significant role in cancer related inflammation and in pregnancy [16–19]. In aggressive human and murine breast cancer cells, a biologically active 20-kDa peptide from the N-terminal domain of a2V (a2NTD) is proteolytically cleaved, secreted in the microvesicles and stimulates human peripheral blood mononuclear cells to produce IL-1β and IL-10 [16–18, 20–25]. Also, our previous *in vitro* and *in vivo* studies showed that a2NTD as well as recombinant a2NTD stimulate M2 polarization of the macrophages, which behave as TAM and promote tumor growth, angiogenesis and invasion in mice [2, 6, 17, 20, 21]. We hypothesized that a2V might regulate neutrophils that can influence breast cancer.

In fact, we show here that a2V is overexpressed on the surface of invasive breast cancer cells. Moreover, in invasive ductal carcinoma (IDC) breast tissues, the increased number of tumor associated neutrophils and blood vessels correlates with the high expression of a2NTD. In addition, a2NTD stimulated human neutrophils (a2Neuɸ) secrete several protumorigenic mediators. a2Neuɸ derived products induce *in vitro* angiogenesis and enhance the invasiveness of breast cancer cells. To our knowledge, this is the first study demonstrating the involvement of a2 isoform V-ATPases in modulating neutrophils by the signal of a2NTD, which in turn promotes tumor progression. These findings demonstrate that a2V and its soluble protein a2NTD could be an important new targets for breast cancer immunotherapy.

## RESULTS

### Expression of a2V and a2NTD in breast cancer cells

**Figure 1:**
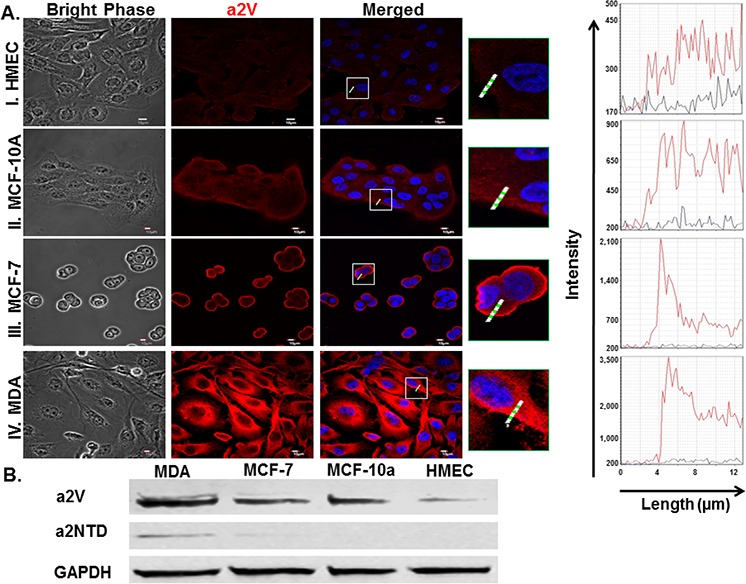
Expression of a2V and a2NTD in breast cancer cells Immunofluorescent analysis of a2V expression **A.** in human non-cancerous mammary epithelial cell lines, HMEC (I.), MCF-10A (II.) and in breast cancer cell lines, MCF-7 (III.), MDA-MB-231 (MDA) (IV.); 2.5 × 10^4^ cells were cultured in 8 well chamber slides, fixed by 4% paraformaldehyde, permeabilized and fluorescently stained by 2c1 antibody (red) and DAPI nuclear staining (blue), examined by confocal microscopy. Histograms represent the Intensity of the staining of the white highlighted region crossing the cells. Original magnification 600 ×; scale bars; 10 μm. Representative images from four independent experiments are shown. B. Cell lysates from the four mammary epithelial cell lines were immunoblotted using 2C1 antibody, anti-a2NTD antibody and GAPDH antibody (as endogenous control) (*n* = 6).

a2V expression in breast cancer cell lines were investigated using immunofluorescence analysis. 2.5 × 10^4^ cells were plated in 8 well chamber slides for overnight to allow the cells to attach to the slide. Cells were fixed, permeabilized and fluorescently stained with anti-a2V (2C1) antibody (red) and DAPI (blue for nuclear staining). Images showed that a2V exhibited a distinctive surface accumulation on invasive breast cancer cell lines MDA-MB-231 (MDA) (highly invasive) and MCF-7 (weakly invasive) as compared to normal human mammary epithelial cell line (HMEC) as well as non-tumorigenic mammary epithelial cells; MCF-10a (Figure 1A). a2V surface expression was positively correlated with cancer cells reported invasiveness. Furthermore, quantitative analysis of the total a2V expression revealed that the highly invasive MDA breast cancer cell line express significantly high levels of a2V (4.5 and 3 fold increase) relative to HMEC and MCF-10A respectively (Supplementary Figure S1A). The total a2V expression was similar in MCF-7 and MCF-10A cells but it differs in its cellular localization; a2V was expressed primarily on the surface of MCF-7 cells however in MCF-10A cells, a2V was primarily cytoplasmic (Figure 1A and Supplementary Figure S1A). Similarly, immunoblotting quantitative analysis confirmed the significant increase of a2V protein levels detected in MDA and MCF-7 breast cancer cell lysates as compared with HMEC cell lysate (Figure 1B and Supplementary Figure S1B). Importantly, a2NTD was detected in the breast cancer cell lysates however it was undetectable in either HMEC or MCF10A (Non-cancerous mammary epithelial cells) (Figure 1B and Supplementary Figure S1C). The increased surface expression of a2V in invasive breast cancer cell lines where a2NTD is detected suggests that a2V together with a2NTD may play an important role in breast cancer progression.

### High levels of a2NTD in breast cancer in relationship with TAN and angiogenesis

**Figure 2:**
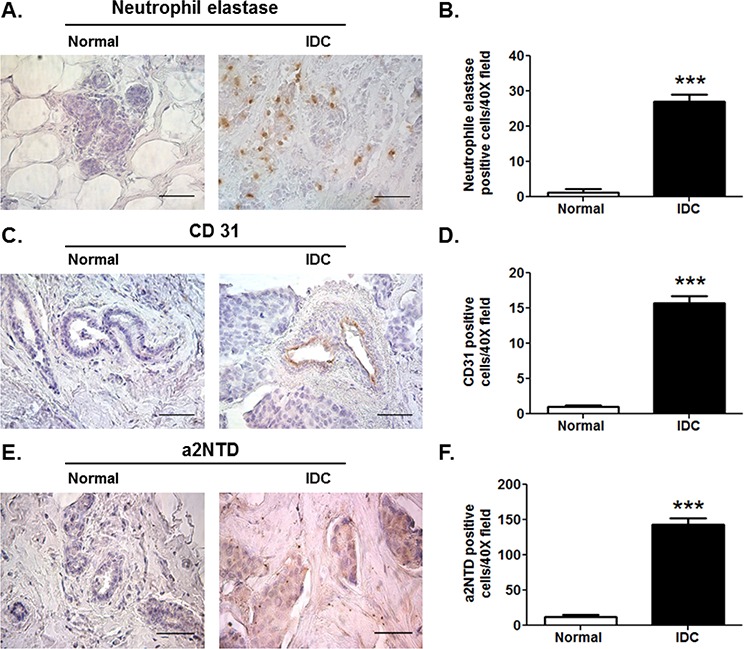
High levels of a2NTD in breast cancer in relationship with TAN and angiogenesis Immunohistochemical staining of neutrophil elastase; neutrophils marker **A.** CD31; endothelial cell marker **C.** and a2NTD **E.** in matched pair-paraffin embedded sections of human normal and infiltrative ductal carcinoma (IDC) breast tissues was examined microscopically. Original magnification 400 ×; scale bars: 50 μm, brown represent positive staining (*n* = 6). Quantification was determined by counting positively stained cells from ten different fields per section **B, D.** and **F.** Data are shown as means ± SEM. ***P < 0.001, as compared with the Normal tissue.

Previous studies show that elevated numbers of neutrophils infiltrating the tumors are positively associated with increased intra-tumoural microvessel density [26, 27]. In order to investigate the correlation between a2NTD expression, TAN infiltration and angiogenesis, immunohistochemical analysis was performed. Increased numbers of recruited neutrophils (27.1 ± 2.02) (Figure 2A and 2B) as well as increased blood vessel density (15.71 ± 1.04) (Figure 2C and 2D) were accompanied by high a2NTD expression (143.2 ± 8.54) (Figure 2E and 2F) in human IDC tissue sections as compared to the adjacent normal tissues (1.28 ± 0.96) (1.1 ± 0.22) and (12.46 ± 2.2), respectively. These results suggest that high a2NTD expression in breast cancer contributes to tumor aggressiveness by modulating neutrophils.

### a2NTD treatment regulates gene and protein expression of pro- and anti-inflammatory cytokines in neutrophils

**Figure 3:**
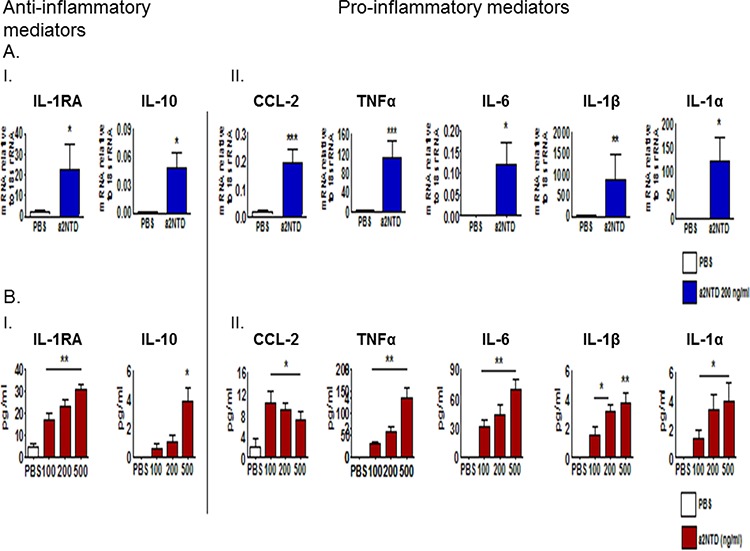
a2NTD treatment regulates gene and protein expression of pro- and anti-inflammatory cytokines in neutrophils Freshly isolated human neutrophils from peripheral blood were suspended in MEM media, treated with either a2NTD 200 ng/ml or PBS as control, incubated for four hours and the mRNA expression **A.** of anti-inflammatory mediators; Interleukin −1 Receptor antagonist; IL-1RA and IL-10 (A.I.) as well as pro-inflammatory mediators (A.II.) in neutrophils were assessed by quantitative real time-PCR. Data were plotted as the mean mRNA expression relative to 18s rRNA ± SEM from at least 3 different experiments. **B.** Neutrophil culture supernatants were collected after 18 hour incubation from a2NTD treated (100, 200 or 500 ng/ml) or PBS treated neutrophils and protein levels of anti- and pro-inflammatory (B.I. or B.II.) mediators were quantified by Multiplex Luminex assay. Data were collected from at least from 4 independent experiments and represented as the mean ± SEM. *P < 0.05, **P < 0.01, ***P < 0.001, as compared with PBS treated neutrophils.

To identify the immunomodulatory effect of the cleaved peptide of a2V (a2NTD) on neutrophils, we used an *in vitro* human model. Quantitative RT-PCR was done to investigate the mRNA expression of several pro- and anti-inflammatory mediators that have been reported previously to be up-regulated in TAN and affect tumor progression [8, 9]. Freshly isolated human neutrophils treated with a2NTD for four hours, showed a significant increase in the relative gene expression of anti-inflammatory mediators such as IL-1RA (38-fold) and IL-10 (11.9-fold) as well as pro-inflammatory cytokines and chemokines such as; TNFα (34.5-fold), IL-1β (94-fold), IL-1α (674-fold), IL-6 (79-fold), CCL-2 (11-fold), CXCL-1 (4.7-fold) and CXCL-2 (31-fold) as compared with the PBS-treated cells (Figure 3AI., 3AII. and Supplementary Figure S2A) (*p* < 0.05). Also, the secreted protein levels of CCL-2, TNFα, IL-6, IL-1β, IL-1α, IL-1RA and IL-10 from a2Neuɸ were detected by multiplex Luminex assay of the neutrophils supernatant collected after 18 hour incubation. Results showed a consistent up-regulation of these mediators in a dose dependent manner except for CCL-2, its secretion decreased upon increasing the a2NTD concentration but the difference between doses was not significant (Figure 3BI. and 3BII.). The mRNA of IL-1β and IL-1α was highly up-regulated, but the secreted protein levels of IL-1β and IL-1α were low. These data demonstrate the contributing effect of a2V in cancer related inflammation by the action of a2NTD on neutrophils.

### a2NTD promotes neutrophils to secret pro-tumorigenic mediators

**Figure 4:**
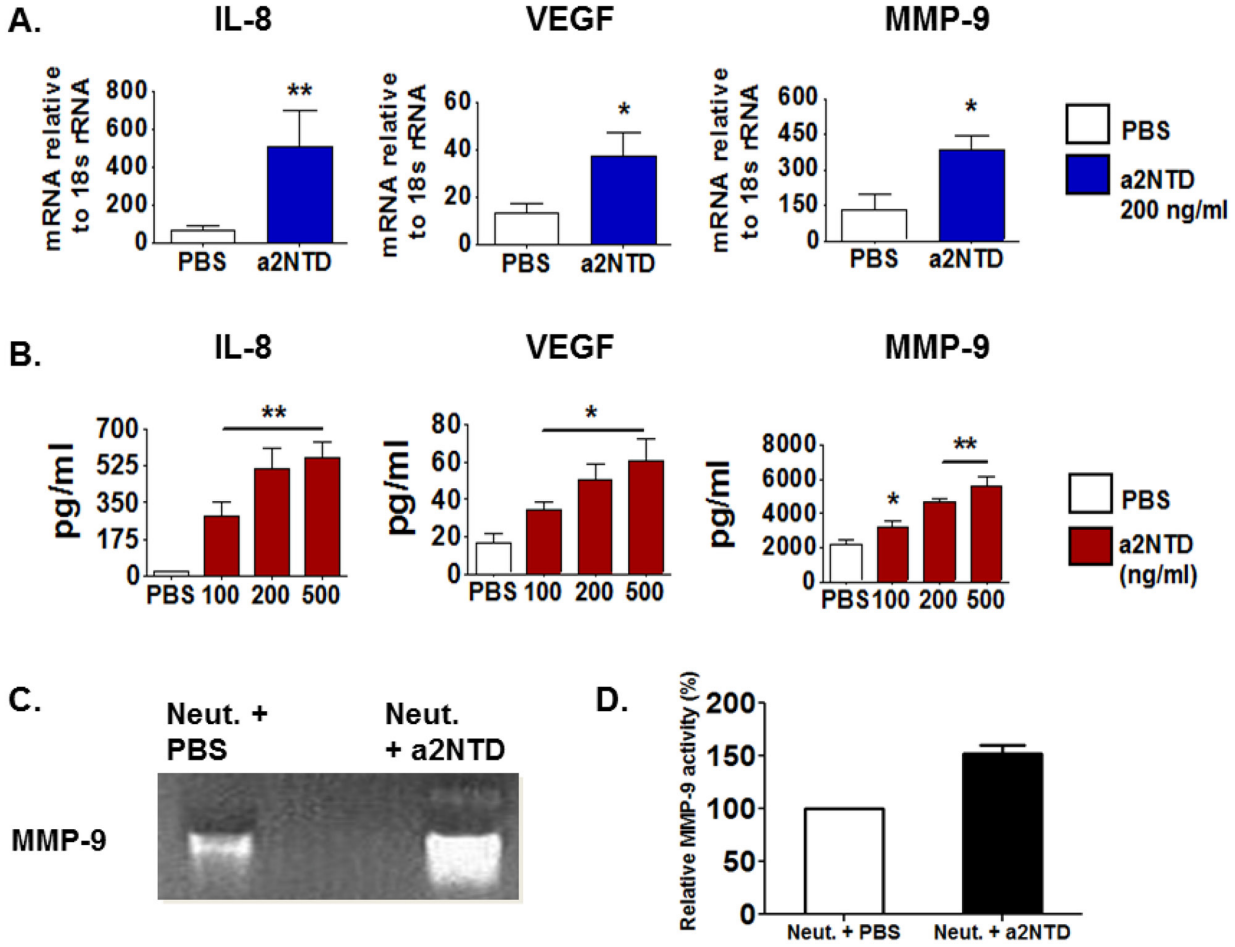
a2NTD promotes neutrophils to secret protumorigenic mediators IL-8, vascular endothelial growth factor (VEGF) and matrix-metalloproteinase-9 (MMP-9) mRNA expression in a2Neuɸ or PBS treated neutrophils were assessed using quantitative real time-PCR **A.** Data were reported as the mean ± SEM, (*n* = 4). The protein levels of IL-8, VEGF and MMP-9 in the neutrophils culture supernatants were quantified using Luminex assay **B.** 10 fold diluted neutrophils culture supernatants were used for MMP-9 quantification. Results were reported as the mean ± SEM, (*n* = 4). *P < 0.05, **P < 0.01, as compared with PBS treated neutrophils. MMP-9 gelatinase activity in neutrophil culture supernatant collected after 18 hour incubation was measured by using a zymogram **C.** The white bands represent the gelatin degraded by MMP-9. Representative data is shown from four individual experiments. Densitometric analysis of the gelatinase activity is showing the percentage of MMP-9 gelatinase activity relative to the PBS-treated control **D.** Results are presented as the mean ± SEM.

The crosstalk between TAN and tumor cells can also stimulate TAN to produce protumorigenic factors that directly affect tumor progression [28]. Quantitative RT-PCR was done to investigate the mRNA expression of several protumorigenic mediators in neutrophils after four hours of a2NTD addition to the culture. a2NTD treatment promotes the mRNA expression of protumorigenic factors correlated with tumor angiogenesis and metastasis; IL-8 (7.98-fold), VEGF (2.2-fold) and MMP-9 (1.87-fold) as compared with PBS-treated neutrophils (Figure 4A).

The secreted protein levels of IL-8, VEGF and MMP-9 were detected by multiplex Luminex assay using neutrophils supernatant collected after overnight incubation with a2NTD. Similarly, the secreted protein levels of these mediators (Figure 4B) were elevated substantially upon a2NTD treatment (IL-8: 561.7 ± 73.2, VEGF; 60.6 ± 11.9, MMP-9; 5,631 ± 529) as compared with PBS-treated neutrophils (IL-8; 19.3 ± 6.1, VEGF; 16.3 ± 5.5, MMP-9; 2,149 ± 209) (*p* < 0.01, *p* < 0.05 for VEGF).

Further, zymographic analysis showed that MMP-9 gelatinase activity was enhanced in a2Neuɸ relative to control (Figure 4C and 4D). In addition, the mRNA expression of other mediators was tested such as SA1009, which showed enhanced up-regulation in a2Neuɸ, however the neutrophil elastase, oncostatin-M, Arginase-1 and SA1008 mRNA expression showed no significant change between PBS-treated neutrophils or a2Neuɸ (Supplementary Figure S2B). Together, these data revealed that a2NTD is not only stimulating neutrophils to secrete specific anti- or pro-inflammatory mediators but also stimulate neutrophils to secrete protumorigenic factors that can directly affect tumor progression.

### a2NTD treated neutrophils induce *in vitro* angiogenesis

TAN are proposed as a key mediator of tumor progression in multiple ways, for instance; promoting angiogenesis which support tumor growth [2, 10, 27, 29]. Since a2Neuɸ secrete substantial amounts of proangiogenic factors, we investigated their functional ability to induce angiogenesis *in vitro*. 1 × 10^4^ HUVEC cells were plated on a 48 well matrigel-coated plate and cultured with either PBS, a2NTD, Neutrophils supernatant or a2Neuɸ supernatant for 48 hours.

**Figure 5:**
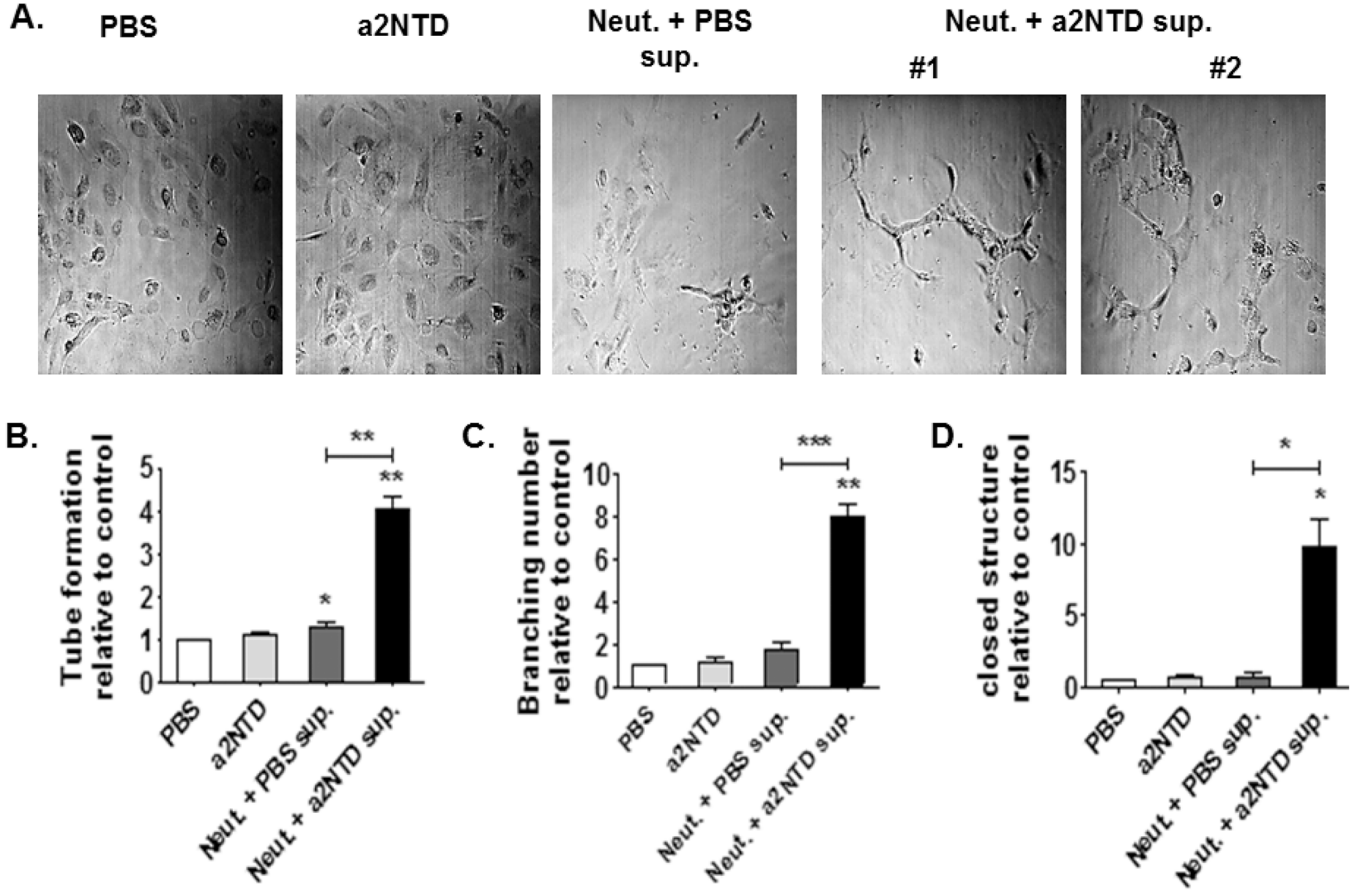
a2NTD treated neutrophils induce *in vitro* angiogenesis A capillary tube formation assay was used to assess angiogenesis *in vitro* **A.** 1 × 10^4^ Human Umbilical cord Endothelial cells (HUVEC) were plated in a 48 well matrigel-coated plate. Cells were cultured with either media + PBS (control), media + a2NTD (a2NTD), neutrophil supernatant (Neut. + PBS sup.) or a2NTD treated neutrophil supernatant (Neut. + a2NTD sup.) for 48 hours. Tube like structures **B.** as well as branching points **C.** and closed structures **D.** were counted from 5 different fields (10 ×) per each well. Data were collected from 4 different experiments each was done in duplicate. Results are reported as the mean ± SEM. *P < 0.05, **P < 0.01, as compared with the control.

Culturing HUVEC cells with a2Neuɸ supernatant induces capillary tube formation, branching of these tubes as well as formation of closed structures by 4, 7 and 9 average fold increases, respectively, as compared to HUVEC cells cultured with PBS-treated neutrophil supernatant (Figure 5A–5D) (*p* < 0.05). On the other hand, a2Neuɸ had no direct effect on human breast cancer cell proliferation (Supplementary Figure S3A and S3B). These data confirmed that a2Neuɸ derived products could promote angiogenesis which may affect tumor growth.

### a2NTD stimulated neutrophils enhance breast cancer cell invasion

**Figure 6:**
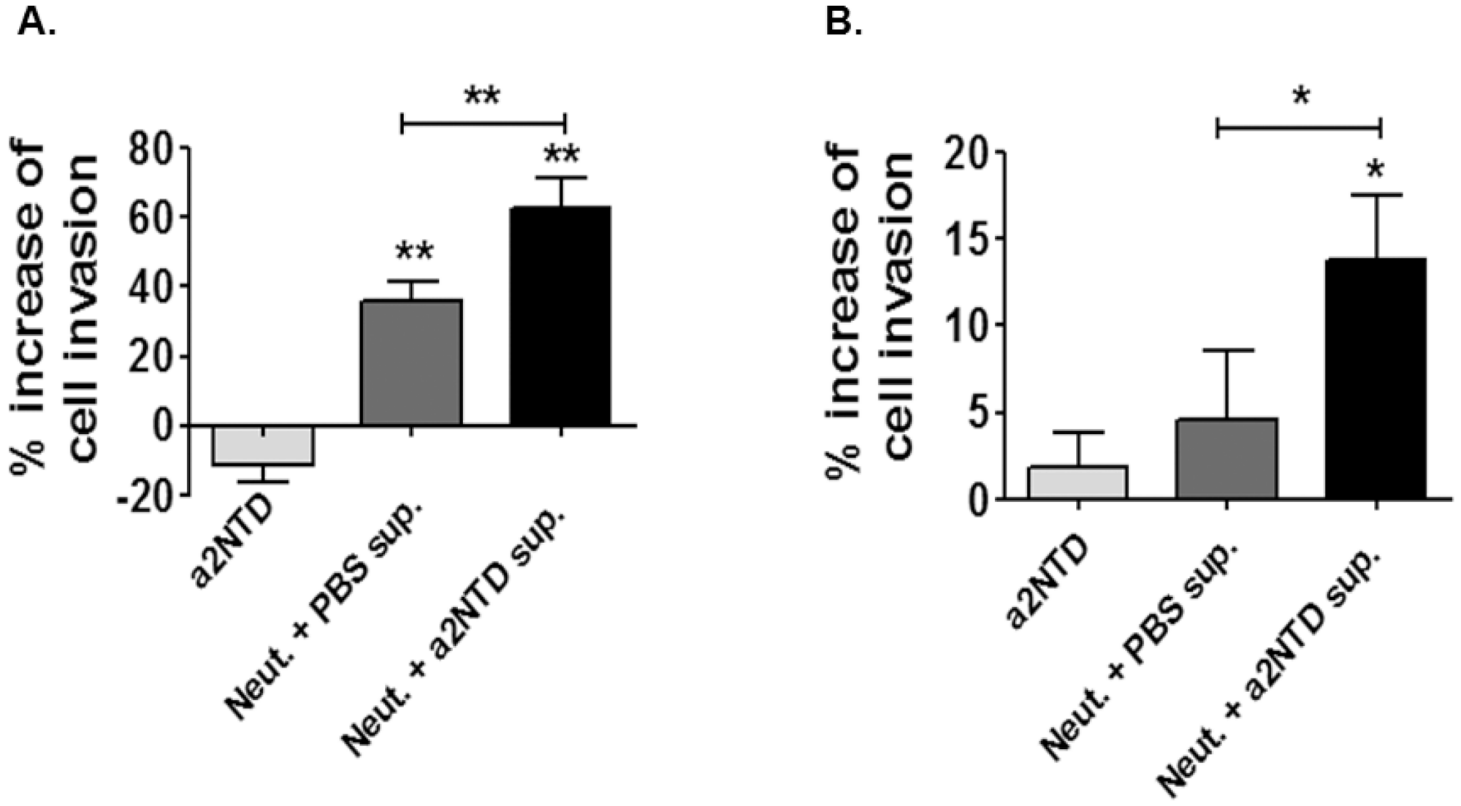
a2NTD stimulated neutrophils enhance breast cancer cell invasion The trans-well invasion assay was carried out on using MDA-MB-231 **A.** and MCF-7 **B.** cell lines. 1 × 10^4^ tumor cells were plated on a basement membrane matrix coated inserts (24 well plates, 8 μm pore size) in serum free media. Tumor cells were either suspended in media + PBS (control), media + a2NTD (a2NTD), neutrophil supernatant (Neut. + PBS sup.) or a2NTD treated neutrophil supernatant (Neut. + a2NTD sup). Results were collected from 3 individual experiments and are reported as the mean of percentage increase in invasion relative to control ± SEM. *P < 0.05, **P < 0.01.

a2Neuɸ secret increased levels of MMP-9; a protease mediating extracellular matrix remodeling which is associated with breast cancer metastasis [30]. To determine whether a2Neuɸ could enhance the invasiveness of breast cancer cells, an *in vitro* transwell invasion assay was performed (Figure 6A and 6B). 1 × 10^4^ tumor cells were plated on a basement membrane matrix coated inserts (24 well plates, 8 μm pore size) in serum free media. Tumor cells were either suspended in the upper chamber with media/PBS (control), media/a2NTD (a2NTD), neutrophil supernatant or a2Neuɸ supernatant.

Culturing MDA and MCF-7 breast cancer cell lines with a2Neuɸ serum free supernatant increases the number of the invading cells compared to cells cultured with either media or PBS-treated neutrophil serum free supernatant (*P* < 0.01 and *P* < 0.05, respectively). Together, our results revealed an important role of a2Neuɸ in promoting breast cancer progression by inducing angiogenesis and enhancing cancer cell invasion.

## DISCUSSION

**Figure 7:**
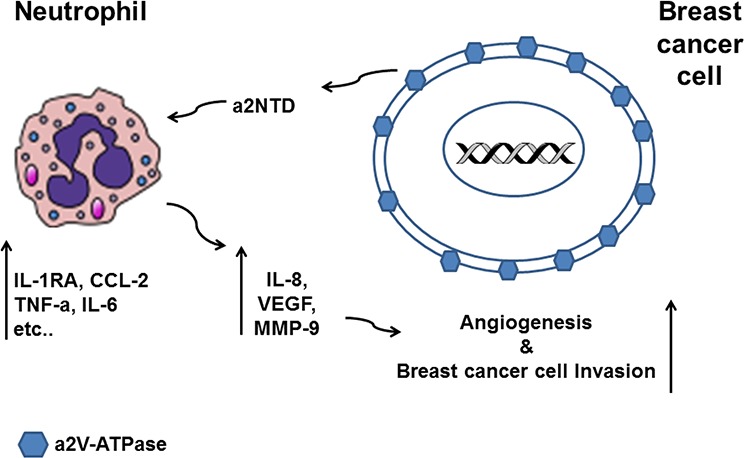
Schematic diagram summarizing the effect of a2NTD treated neutrophils on breast cancer progression Breast cancer cells overexpress a2V-ATPase on the surface and secrete a2NTD that regulates the gene expression of the neutrophils. a2NTD stimulates neutrophils to synthesize and secret increased levels of the protumorigenic factors such as IL-8, VEGF and MMP-9. a2Neuɸ derived products induce angiogenesis and promote the invasive potential of human breast cancer cells.

The important role of neutrophils in cancer biology is well established [2]. Especially, their contribution to tumor angiogenesis and invasive growth has become increasingly clear [7, 26]. Thus, identifying the factors that modulate neutrophils in the tumor site is important for the development of targeted immunotherapy. Here we show a novel mode of regulating TAN through the action of tumor associated V-ATPases. We present that a2V and its soluble protein a2NTD are highly expressed in breast cancer. In addition, increased neutrophils recruitment to breast tumor site was associated with elevated a2NTD expression as well as increased angiogenesis marker. We also show the direct effect of a2NTD on promoting the protumorigenic properties of neutrophils by regulating their gene expression and stimulating the secretion of protumoral mediators, which was associated with the induction of angiogenesis and the enhancement of the invasiveness of human breast cancer cells (Figure 7). Therefore, we have identified a new role of the tumor associated immunoregulatory molecule; a2NTD that can modulate neutrophils, an important member of the innate immunity in tumor microenvironment.

V-ATPases are involved in cancer initiation as well as cancer progression [31, 32]. Recent reports showed that functional inhibition or siRNA targeting of specific V-ATPases can significantly reduce tumor growth, metastasis and overcome chemoresistance in breast cancer [12–15, 33]. Apart from the direct role of a2V in tumor progression by its ability to regulate pH and invasion, it also has immunomodulatory role in the tumor microenvironment through its released peptide a2NTD [21, 34, 35]. We have shown before that a2NTD skewed monocyte differentiation toward the TAM phenotype, which support tumor growth, angiogenesis and invasion [6, 16, 17, 21]. In this study, we showed that a2V was highly expressed on the surface of human breast cancer cell lines and was associated with the detection of a2NTD in their cell lysates. On the other hand, in non-cancerous mammary epithelial cell lines, a2NTD was undetectable and a2V expression was cytoplasmic compared to breast cancer cells. Interestingly, the a2V cellular localization was different among the different subtypes of breast cancer cell lines. MCF-7 cells, a luminal A subtype (positive for Estrogen (ER) and progesterone (PR) receptor) exhibited high a2V membrane staining and low cytoplasmic staining. On the other hand, MDA-MB-231 and MCF-10A cells, basal-like subtype (ER, PR, HER2 negative and EGFR positive) showed more cytoplasmic staining. It is well known that the breast cancer basal-like subtype is more aggressive than the Luminal subtype and is more frequently associated with metastasis [36, 37]. This suggests that the high expression of the cytoplasmic as well as the membranous a2V in the MDA-MB-231 might be correlated with the high aggressiveness of this cell line. Together, these data suggest that high surface expression of a2V on cancer cells and its soluble peptide a2NTD may have potential role in breast cancer progression.

Inflammatory cells represent a significant member of cells seen in various types of tumors [38, 39]. Although, neutrophils are short living cells in blood circulation, they survive much longer under inflammatory conditions [40]. The continuous recruitment of neutrophils to the tumor site in response to the presence of neutrophil chemoattractants such as IL-8, CXCL-1 and CXCL-2 in the tumor microenvironment, make TAN capable of regulating tumor growth and metastasis [5, 28]. In accordance, TAN are considered as an independent prognostic marker in a wide variety of cancer [2]. For instance; elevated neutrophils recruitment is associated with poor clinical outcomes in bronchioalveolar carcinoma and renal cell carcinoma [41, 42]. Similarly, it is associated with the aggressive types of pancreatic neoplasms [43]. In human breast cancer, neutrophils infiltration was correlated positively with increased blood vessel density as detected by high CD31 expression (endothelial cell marker) [26]. Similarly, we showed that in IDC breast cancer tissues, the increased numbers of infiltrated neutrophils was accompanied by high blood vessel density along with increased expression of a2NTD as compared with the adjacent normal tissues. Since factors modulating TAN behavior in breast cancer remain unclear, these data made a2NTD as a potential signaling molecule which can regulate neutrophils in tumor site.

A transcriptomic analysis showed that TAN overexpress several cytokines and chemokines that are known to contribute to cancer related inflammation [8, 9]. In this study, we examined the effect of a2NTD on neutrophils gene expression of these mediators in different time points and we found that the earliest response was detected after four hours of adding a2NTD to the culture and this effect increased with time. This early response indicates that a2NTD was the only factor driving these changes in neutrophils gene expression without other interfering factors. In addition, we confirmed these changes in neutrophils gene expression by examining the secreted levels of different mediators after overnight culture to insure sufficient time for the translation and the secretion of these mediators.

Our data demonstrates that a2NTD treatment stimulates neutrophils to overexpress and enhance the secretion of anti-inflammatory mediators such as IL-1RA and IL-10; important mediators in regulating the immune response in tumor site. In addition, the elevated expression of pro-inflammatory cytokines such as TNFα, IL-1 and IL-6 in breast cancer is directly correlated with the metastatic behavior and progression of breast carcinomas [44–48]. In our model, a2NTD treatment stimulates neutrophils to secrete enhanced amount of TNFα, IL-1 and IL-6 suggesting that neutrophils in the site of the tumor may be a potential source of these mediators. Similarly, a2NTD treatment enhanced the expression of different chemokines, For instance; CXCL-1, CXCL-2 and IL-8, which are a potent neutrophil chemoattractants, CCL-2; a monocytes chemoattractant, suggesting that a2NTD programmed neutrophils might be a part of a paracrine network in the tumor site to insure a constant recruitment of neutrophils and monocytes to the site of the tumor, in order to promote tumor progression. Further *in vivo* studies will confirm this possibility. Together, these data demonstrate the immunomodulatory effect of a2NTD on neutrophils to secrete several mediators similar to that secreted by TAN. This finding reflects the contributing role of a2V in cancer related inflammation by regulating neutrophils.

The interaction between TAN and tumor cells can also stimulate TAN to produce pro-tumorigenic factors that directly affect tumor progression [28]. TAN promote angiogenesis and metastasis in tumors by secreting protumorigenic factors, for instance; IL-8 and VEGF, potent pro-angiogenic mediators as well as MMP-9 to degrade extracellular matrix, which promote tumor growth, angiogenesis and metastasis [9, 10, 27, 28]. Uniquely, human neutrophils secrete tissue inhibitor of metalloproteinases (TIMP)-free MMP-9 which provides a potent catalytic stimulator of angiogenesis [27, 49]. In addition, TAN are considered to be the main source of MMP-9 in several types of tumors [10, 29, 50, 51]. Here, we show that IL-8, VEGF and MMP-9 mRNA were up-regulated in a2Neuɸ. Consistently, a2NTD stimulates substantial quantities of these protumoral mediators to be secreted from neutrophils. Further, MMP-9 activity was enhanced significantly upon a2NTD treatment. Knowing the signaling pathways involved in these significant changes that occurred in the neutrophils after a2NTD addition, will provide us with better understanding on how to develop proper anticancer therapies. Interestingly, several key target genes of NFkB pathway were upregulated significantly in neutrophils upon a2NTD treatment such as, CCL-2, TNF-α, IL-1β, IL-8 and MMP-9 (shown in Figure 3 and 4). This effect Suggests that a2NTD treatment lead to NFkB activation and further studies are designed to further investigate this effect and also to study the effect of a2NTD on other signaling pathways.

Taken together, we speculate that a2Neuɸ secreted products affect tumor growth, angiogenesis and invasion. We showed that a2Neuɸ secreted products significantly induce angiogenesis *in vitro* and increase the invasiveness of the human breast cancer cells. Since the formation of new blood vessels is crucial for nourishing the tumor as well as facilitating the tumor metastasis [30, 25], we believe that a2Neuɸ support breast cancer growth and progression by enhancing angiogenesis and breast cancer cell invasiveness.

In summary, this study demonstrates the significant role that V-ATPase can play in breast cancer progression and demonstrates a link between tumor associated a2V and innate immunity. We provide evidence that breast cancer associated a2V promotes the protumorigenic characteristics in neutrophils by the action of a2NTD, which supports tumor progression. This suggests that a2V and its cleaved peptide a2NTD can be potential targets for breast cancer therapy. Further investigation into the mechanisms involved in neutrophil a2NTD uptake and downstream processes are warranted to clarify our understanding of the link between a2V and cancer related inflammation in breast cancer.

## MATERIALS AND METHODS

### Cells and tissue samples

These studies were approved by the Rosalind Franklin University of Medicine and Science Institutional Review Board. After informed consent was obtained in accordance with the Declaration of Helsinki, peripheral blood was collected from healthy adult volunteers into sodium heparin vacutainers (Thermo Fisher Scientific Inc., Waltham, MA, USA). Neutrophils were isolated using the dextran-Ficoll technique under endotoxin-free conditions using Ficoll-Paque PLUS (Thermo Fisher) as described previously [52]. The neutrophil cell pellet was, re-suspended in MEM complete media (Invitrogen, Grand Island, NY, USA) containing 10% heat inactivated fetal bovine serum (FBS), penicillin (100 U/ml), streptomycin sulfate (100 mg/ml), and 2.0 mM glutamine. Neutrophils purity was 97–99% (CD15/high side scatter) with 99% viability as determined by flowcytometry (AnnexinV/7-AAD^−/−^) and trypan blue exclusion. Three human mammary epithelial cell lines; invasive breast cancer cell lines MCF-7 (ATCC, HTB-22), MDA-MB-231 (MDA) (ATCC, HTB-26), non-tumorigenic mammary gland epithelial cells MCF10a (ATCC, CRL-10317) were purchased from the American Type Culture Collection (ATTC, Manassas, VA, USA). Normal human mammary epithelial cell line HMEC was purchased from Cell Applications Inc., San Diego, CA. Human umbilical vein endothelial cells (HUVEC) were a gift from Dr. Bala Chandran. MDA and MCF-7 cell lines were cultured in MEM complete medium (Invitrogen). For the MCF-7 cell line, 0.01 percent insulin (Sigma, I9278-5ML) was added to the culture media. MCF-10a was cultured in complete MEBM (Lonza/Clonetics, USA) as recommended by ATCC. HMEC cell line was maintained in MEGM media (Cell Applications). HUVEC cell line was cultured in EBM (Lonza) supplemented with EGM SingleQuots Kit (Lonza). Cells were cultured at 37°C in a humidified atmosphere containing 5% (v/v) CO_2_. Cells were grown until reaching approximately 80% confluence and then subcultured or plated for experiments. For immunohistochemistry Paraffin embedded human tissue sections of 5-μm size from breast infiltrative ductal carcinomas or adjacent normal tissues were procured from Biochain, Newark, CA, USA.

### Immunofluorescence analysis

Breast Cell lines were cultured in 8 well chamber slides, fixed, permeabilized and stained with anti-a2V (2C1) (mouse monoclonal antibody to a2V), was generated as previously described [34] specific for aa 488–510, the transmembrane region of the protein. Alexa Fluor® 594-conjugated donkey anti-mouse secondary antibody (1:200 dilution) (Invitrogen) was used. The cells were prepared for viewing using ProLong® Gold (Invitrogen) mounting medium containing DAPI. Stained cell lines were imaged by an Olympus Fluoview FV10i confocal microscope and analyzed by FV10i Fluoview Ver.3.0 software.

### Immunoblotting

Equal amounts of total protein lysates were mixed with sample buffer, heated at 95°C for 5 min and loaded to SDS-PAGE and transferred to nitrocellulose. The membranes were blocked in protein-free PBS blocking buffer (Thermo Scientific, Rockford, IL) for 1 h at room temperature. Primary and secondary antibodies were incubated in protein-free blocking buffer/0.05% Tween- 20 for 1 h at room temperature. Protein signals were detected using an Odyssey imaging instrument and analyzed using instrument software (Li-Cor Biosciences). The intensities of the immunoreactive bands were quantified by densitometric analysis using ImageJ software.

### Immunohistochemical staining of human breast tissue

Immunohistochemical staining of Paraffin embedded human tissue sections of 5-μm size from breast infiltrative ductal carcinomas (IDC) or adjacent normal tissues collected from six different donors was performed using Dako EnVision™+ Dual Link System-HRP (Dako, Carpinteria, CA) system according to manufacturer`s protocol. anti-a2NTD (antibody to a2NTD) was generated as previously described [16], specific for aa 142–344, the N-terminal region of the a2 subunit, anti-human CD31 JC70A (Dako) and anti-human neutrophil elastase NP-57(Santa Cruz biotechnology). Heat-induced epitope retrieval in either sodium citrate buffer pH = 6 (for a2NTD detection [19]) or in Tris-EDTA buffer pH = 9 (For CD31 detection [53]) preceded the primary antibody incubation. For controls, tissue sections were stained with isotype-control antibodies (R&D systems, USA) using at the same concentration as the primary antibodies. Immunostaining was evaluated by light photomicroscopy (Carl Zeiss, Weesp, The Netherlands) using a high-resolution camera (Canon G10, Canon, Tokyo, Japan). Scale bars were calculated using ImageJ software. Quantification was determined by counting 10 different 400× fields per tissue section.

### Recombinant a2NTD

a2NTD was expressed and purified from *Escherichia coli* and subjected to endotoxin removal column chromatography (Proteome Resources, Aurora, CO) as previously described [17, 20].

### RNA isolation and real time PCR

After treating neutrophils with PBS (control) or recombinant a2NTD for 4 hr incubation, total RNA was isolated using RNeasy micro kit (Qiagen, Germantown, MD) and single-stranded cDNA was synthesized using QuantiTect reverse transcription kit (Qiagen). Qantitative real-time PCR (Q RT-PCR) was performed, using cDNA specific FAM-MGB–labeled Taqman primer sets (Applied Biosystems) for various genes and VIC-MGB labeled *18s rRNA* was used as endogenous control.

### Cytokine/Chemokine and MMP-9 bioassay

The secretion of a panel of Human cytokine/chemokines and MMP-9 was analyzed by Milliplex map kit (Millipore, St. Charles, MO) in the supernatant of neutrophils (1 × 10^6^ cell/ml) collected after overnight incubation, and assayed on a MAGPIX instrument (Millipore) as per the instructions provided by manufacture. Equal volumes from cell supernatant were used for the assay. The assay was performed for six independent experiments.

### Gelatin zymography

Equal volumes of Neutrophil supernatant were mixed with sample buffer (2 ×) and applied directly to 10% zymogram gels (Invitrogen) containing 1 mg/ml gelatin without prior heating or reduction. SDS was removed from the gel by incubating in 2.5% (v/v) Triton X-100 (Fisher Scientific) for 30 min. The gels were kept at 37°C in development buffer (50 mmol/l Tris-HCl, pH 7.6, containing 0.2 m NaCl, 5 mmol/l CaCl2) for 3 h at 37°C. Gels were then stained with 0.2% Coomassie blue. White bands representing gelatinase activity were photographed in gel documentation system (Biorad).

### *In vitro* angiogenesis assay

HUVEC cells (1 × 10^4^ cells/well) were seeded into a BD Matrigel (BD Biosciences, San Jose, CA, USA) coated (100 μl/well) 48-well plate and 200 μl of PBS treated neutrophil supernatant, a2NTD treated neutrophil supernatant or their respective controls were added to assigned wells, and incubated for 48 h at 37°C. Tube-like structures, branching and closed structures of HUVEC formed at 48 h were counted from 5 different fields by a microscope.

### *In vitro* invasion assay

The invasion assay was conducted using the CytoSelect 24-well cell invasion assay kit (Cell Biolabs Inc, San Diego, CA, USA). Briefly, MDA or MCF-7 cells (1 × 10^4^ cells/insert) were added to 250 μl of serum-free PBS treated (control) or a2NTD treated neutrophil supernatant or relative controls in the upper chambers. MEM (500 μl) with 10% FBS was added to the lower chamber. After 17 h of incubation, invaded cells were quantified using fluorometric analysis according to the manufacturer's instructions.

### Statistical analysis

Statistical analysis was performed with Mann–Whitney test or paired *t*-test using Graph Pad Prism 5 (GraphPad Software, Inc., San Diego, CA, USA). Error bars show means ± SEM. *P* value less than 0.05 was considered significantly different.

## SUPPLEMENTARY FIGURES



## References

[1] Cortez-Retamozo V, Etzrodt M, Newton A, Rauch PJ, Chudnovskiy A, Berger C (2012). Origins of tumor-associated macrophages and neutrophils. Proc. Natl. Acad. Sci.

[2] Mantovani A (2014). Macrophages, Neutrophils, and Cancer: A Double Edged Sword. New J. Sci.

[3] Queen MM, Ryan RE, Holzer RG, Keller-Peck CR, Jorcyk CL (2005). Breast cancer cells stimulate neutrophils to produce oncostatin M: potential implications for tumor progression. Cancer Res.

[4] Swierczak A (2012). The role of myeloid cells in breast cancer metastasis.

[5] Acharyya S, Oskarsson T, Vanharanta S, Malladi S, Kim J, Morris PG (2012). A CXCL1 paracrine network links cancer chemoresistance and metastasis. Cell.

[6] Katara GK, Jaiswal MK, Kulshrestha A, Kolli B, GilmanSachs A, Beaman KD (2014). Tumor-associated vacuolar ATPase subunit promotes tumorigenic characteristics in macrophages. Oncogene.

[7] Fridlender ZG, Sun J, Kim S, Kapoor V, Cheng G, Ling L (2009). Polarization of tumor-associated neutrophil phenotype by TGF-β:“N1” versus “N2” TAN. Cancer Cell.

[8] Eruslanov EB, Bhojnagarwala PS, Quatromoni JG, Stephen TL, Ranganathan A, Deshpande C (2014). Tumor-associated neutrophils stimulate T cell responses in early-stage human lung cancer. J. Clin. Invest.

[9] Fridlender ZG, Sun J, Mishalian I, Singhal S, Cheng G, Kapoor V (2012). Transcriptomic analysis comparing tumor-associated neutrophils with granulocytic myeloid-derived suppressor cells and normal neutrophils. PloS One.

[10] Kuang D-M, Zhao Q, Wu Y, Peng C, Wang J, Xu Z (2011). Peritumoral neutrophils link inflammatory response to disease progression by fostering angiogenesis in hepatocellular carcinoma. J. Hepatol.

[11] Sennoune SR, Bakunts K, Martínez GM, Chua-Tuan JL, Kebir Y, Attaya MN (2004). Vacuolar H+-ATPase in human breast cancer cells with distinct metastatic potential: distribution and functional activity. Am. J. Physiol.-Cell Physiol.

[12] Feng S, Zhu G, McConnell M, Deng L, Zhao Q, Wu M (2013). Silencing of atp6v1c1 prevents breast cancer growth and bone metastasis. Int. J. Biol. Sci.

[13] You H, Jin J, Shu H, Yu B, De Milito A, Lozupone F (2009). Small interfering RNA targeting the subunit ATP6L of proton pump V-ATPase overcomes chemoresistance of breast cancer cells. Cancer Lett.

[14] Von Schwarzenberg K, Lajtos T, Simon L, Müller R, Vereb G, Vollmar AM (2014). V-ATPase inhibition overcomes trastuzumab resistance in breast cancer. Mol. Oncol.

[15] Wiedmann RM, von Schwarzenberg K, Palamidessi A, Schreiner L, Kubisch R, Liebl J (2012). The V-ATPase-inhibitor archazolid abrogates tumor metastasis via inhibition of endocytic activation of the Rho-GTPase Rac1. Cancer Res.

[16] Ntrivalas E, Derks R, Gilman-Sachs A, Kwak-Kim J, Levine R, Beaman K (2007). Novel role for the N-terminus domain of the a2 isoform of vacuolar ATPase in interleukin-1β production. Hum. Immunol.

[17] Kwong C, Gilman-Sachs A, Beaman K (2011). Tumor-associated a2 vacuolar ATPase acts as a key mediator of cancer-related inflammation by inducing pro-tumorigenic properties in monocytes. J. Immunol.

[18] Kwong C, Gilman-Sachs A, Beaman K (2013). An independent endocytic pathway stimulates different monocyte subsets by the a2 N-terminus domain of vacuolar-ATPase. Oncoimmunology.

[19] Jaiswal MK, Mallers TM, Larsen B, Kwak-Kim J, Chaouat G, Gilman-Sachs A (2012). V-ATPase upregulation during early pregnancy: a possible link to establishment of an inflammatory response during preimplantation period of pregnancy. Reproduction.

[20] Ntrivalas E, Gilman-Sachs A, Kwak-Kim J, Beaman K (2007). The N-terminus Domain of the a2 Isoform of Vacuolar ATPase Can Regulate Interleukin-1β Production from Mononuclear Cells in Co-culture with JEG-3 Choriocarcinoma Cells. Am. J. Reprod. Immunol.

[21] Katara GK, Kulshrestha A, Jaiswal MK, Pamarthy S, Gilman-Sachs A, Beaman KD (2015). Inhibition of vacuolar ATPase subunit in tumor cells delays tumor growth by decreasing the essential macrophage population in the tumor microenvironment. Oncogene.

[22] Mandal M, Beaman KD (1995). Purification and Characterization of a Pregnancy-Associated Protein: TJ6s. Am. J. Reprod. Immunol.

[23] Beaman K, Angkachatchai V, Gilman-Sachs A (1996). TJ6: The Pregnancy-Associated Cytokine. Am. J. Reprod. Immunol.

[24] Lee GW, Boomer JS, Gilman-Sachs A, Chedid A, Gudelj L, Rukavina D (2001). Regeneration and tolerance factor of the human placenta induces IL-10 production. Eur. J. Immunol.

[25] Roth P, Aulwurm S, Gekel I, Beier D, Sperry RG, Mittelbronn M (2006). Regeneration and tolerance factor: a novel mediator of glioblastoma-associated immunosuppression. Cancer Res.

[26] Jubb AM, Soilleux EJ, Turley H, Steers G, Parker A, Low I (2010). Expression of vascular notch ligand delta-like 4 and inflammatory markers in breast cancer. Am. J. Pathol.

[27] Tazzyman S, Lewis CE, Murdoch C (2009). Neutrophils: key mediators of tumour angiogenesis. Int. J. Exp. Pathol.

[28] Gregory AD, Houghton AM (2011). Tumor-associated neutrophils: new targets for cancer therapy. Cancer Res.

[29] Nozawa H, Chiu C, Hanahan D (2006). Infiltrating neutrophils mediate the initial angiogenic switch in a mouse model of multistage carcinogenesis. Proc. Natl. Acad. Sci.

[30] Wu Q-W, Yang Q-M, Huang Y-F, She H-Q, Liang J, Yang Q-L (2014). Expression and clinical significance of matrix metalloproteinase-9 in lymphatic invasiveness and metastasis of breast cancer. PloS One.

[31] Lu X, Qin W (2012). Deep Insight Section. HttpAtlasGenetics Oncology Org.

[32] Fais S, De Milito A, You H, Qin W (2007). Targeting vacuolar H+-ATPases as a new strategy against cancer. Cancer Res.

[33] Von Schwarzenberg K, Wiedmann RM, Oak P, Schulz S, Zischka H, Wanner G (2013). Mode of cell death induction by pharmacological vacuolar H+-ATPase (V-ATPase) inhibition. J. Biol. Chem.

[34] Derks R, Beaman K (2004). Regeneration and tolerance factor modulates the effect of adenosine triphosphate–induced interleukin 1β secretion in human macrophages. Hum. Immunol.

[35] Kulshrestha A, Katara GK, Ibrahim S, Pamarthy S, Jaiswal MK, Sachs AG (2015). Vacuolar ATPase “a2” isoform exhibits distinct cell surface accumulation and modulates matrix metalloproteinase activity in ovarian cancer. Oncotarget.

[36] Subik K, Lee J-F, Baxter L, Strzepek T, Costello D, Crowley P (2010). The expression patterns of ER, PR, HER2, CK5/6, EGFR, Ki-67 and AR by immunohistochemical analysis in breast cancer cell lines. Breast Cancer Basic Clin. Res.

[37] Kennecke H, Yerushalmi R, Woods R, Cheang MCU, Voduc D, Speers CH (2010). Metastatic behavior of breast cancer subtypes. J. Clin. Oncol.

[38] Mantovani A, Allavena P, Sica A, Balkwill F (2008). Cancer-related inflammation. Nature.

[39] Johansson M, DeNardo DG, Coussens LM (2008). Polarized immune responses differentially regulate cancer development. Immunol. Rev.

[40] Mantovani A (2009). The yin-yang of tumor-associated neutrophils. Cancer Cell.

[41] Wislez M, Rabbe N, Marchal J, Milleron B, Crestani B, Mayaud C (2003). Hepatocyte growth factor production by neutrophils infiltrating bronchioloalveolar subtype pulmonary adenocarcinoma role in tumor progression and death. Cancer Res.

[42] Jensen HK, Donskov F, Marcussen N, Nordsmark M, Lundbeck F, von der Maase H (2009). Presence of intratumoral neutrophils is an independent prognostic factor in localized renal cell carcinoma. J. Clin. Oncol.

[43] Reid MD, Basturk O, Thirabanjasak D, Hruban RH, Klimstra DS, Bagci P (2011). Tumor-infiltrating neutrophils in pancreatic neoplasia. Mod. Pathol.

[44] Ben-Baruch A (2002). Host microenvironment in breast cancer development: Inflammatory cells, cytokines and chemokines in breast cancer progression-reciprocal tumor–microenvironment interactions. Breast Cancer Res.

[45] Leek RD, Landers R, Fox SB, Ng F, Harris AL, Lewis CE (1998). Association of tumour necrosis factor alpha and its receptors with thymidine phosphorylase expression in invasive breast carcinoma. Br. J. Cancer.

[46] Madhusudan S, Foster M, Muthuramalingam SR, Braybrooke JP, Wilner S, Kaur K (2004). A phase II study of etanercept (Enbrel), a tumor necrosis factor α inhibitor in patients with metastatic breast cancer. Clin. Cancer Res.

[47] Kurebayashi J (2000). Regulation of interleukin-6 secretion from breast cancer cells and its clinical implications. Breast Cancer.

[48] Jin L, Yuan RQ, Fuchs A, Yao Y, Joseph A, Schwall R (1997). Expression of interleukin-1β in human breast carcinoma. Cancer.

[49] Ardi VC, Kupriyanova TA, Deryugina EI, Quigley JP (2007). Human neutrophils uniquely release TIMP-free MMP-9 to provide a potent catalytic stimulator of angiogenesis. Proc. Natl. Acad. Sci.

[50] Shang K, Bai Y-P, Wang C, Wang Z, Gu H-Y, Du X (2012). Crucial involvement of tumor-associated neutrophils in the regulation of chronic colitis-associated carcinogenesis in mice. PLoS One.

[51] Coussens LM, Tinkle CL, Hanahan D, Werb Z (2000). MMP-9 supplied by bone marrow–derived cells contributes to skin carcinogenesis. Cell.

[52] Hirota Y, Osuga Y, Hirata T, Harada M, Morimoto C, Yoshino O (2005). Activation of protease-activated receptor 2 stimulates proliferation and interleukin (IL)-6 and IL-8 secretion of endometriotic stromal cells. Hum. Reprod.

[53] Righi L, Deaglio S, Pecchioni C, Gregorini A, Horenstein AL, Bussolati G (2003). Role of CD31/platelet endothelial cell adhesion molecule-1 expression in *in vitro* and *in vivo* growth and differentiation of human breast cancer cells. Am. J. Pathol.

