# Supplementary material for: Breast cancer associated a2 isoform vacuolar ATPase immunomodulates neutrophils: potential role in tumor progression
Source: Oncotarget. 2015 Oct 9;6(32):33033–45. doi: 10.18632/oncotarget.5439 (PMC4741747; doi:10.18632/oncotarget.5439)
Supplement: Supplementary file 1 [file oncotarget-06-33033-s001.pdf]

SUPPLEMENTARY FIGURES

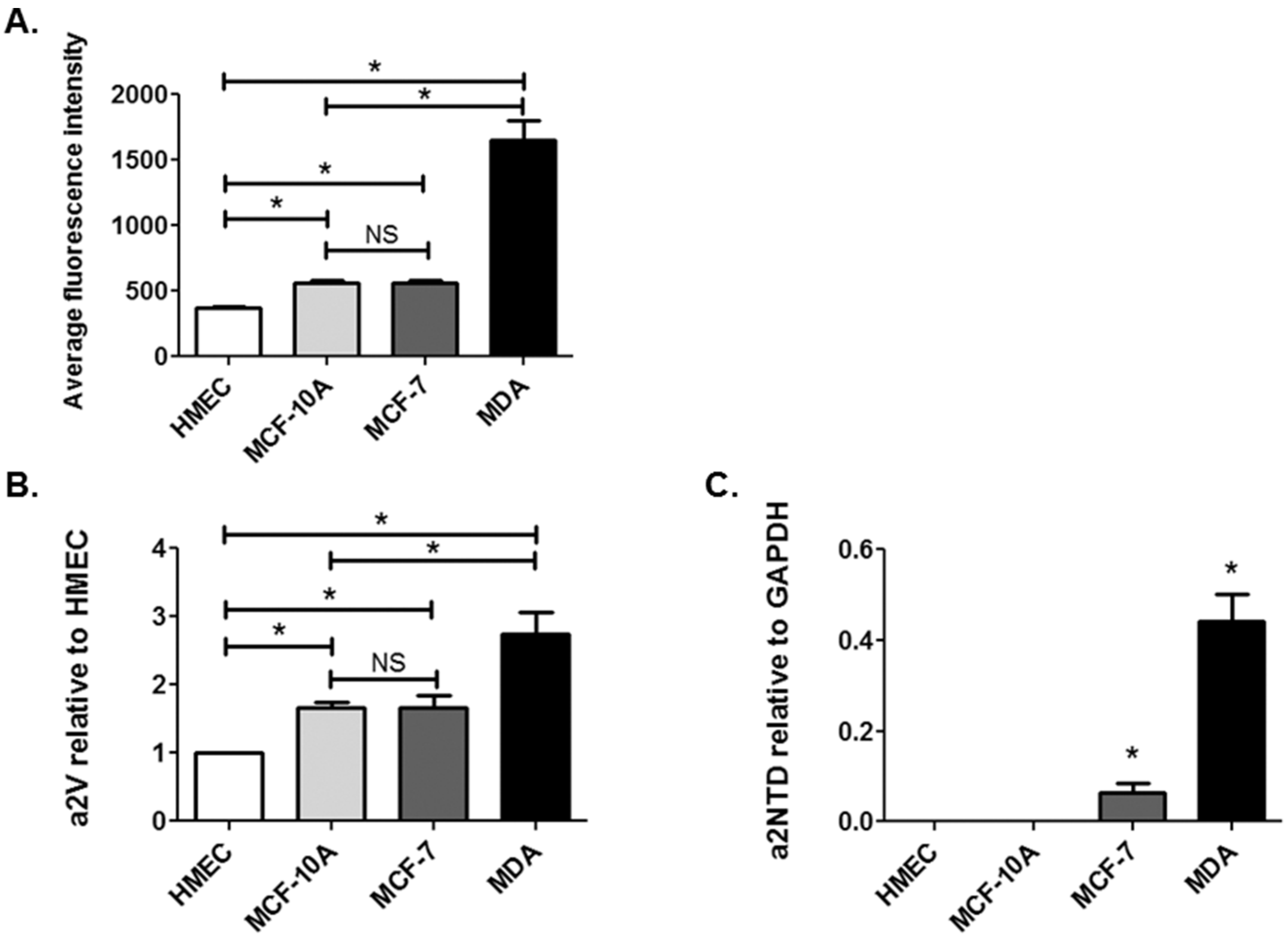

Supplementary Figure S1: Quantitative analysis of a2V and a2NTD expression in breast cancer cells. A. Quatification of the intensity of the immunofluorescent a2V staining in HMEC, MCF-10A and in breast cancer cell lines, MCF-7 and MDA shown in Figure 1A was performed using FV10i Fluoview Ver.3.0 software ($n = 4$). Data are shown as means ± SEM. *$P < 0.05$, as compared with HMEC or MCF-10A. **B.** and **C.** Intensities of immunoreactive bands on Western blots shown in Figure 1B were quantified by densitometric analysis using ImageJ software ($n = 6$). Data are presented as means ± SEM. *$P < 0.05$, as compared with HMEC or MCF10A.

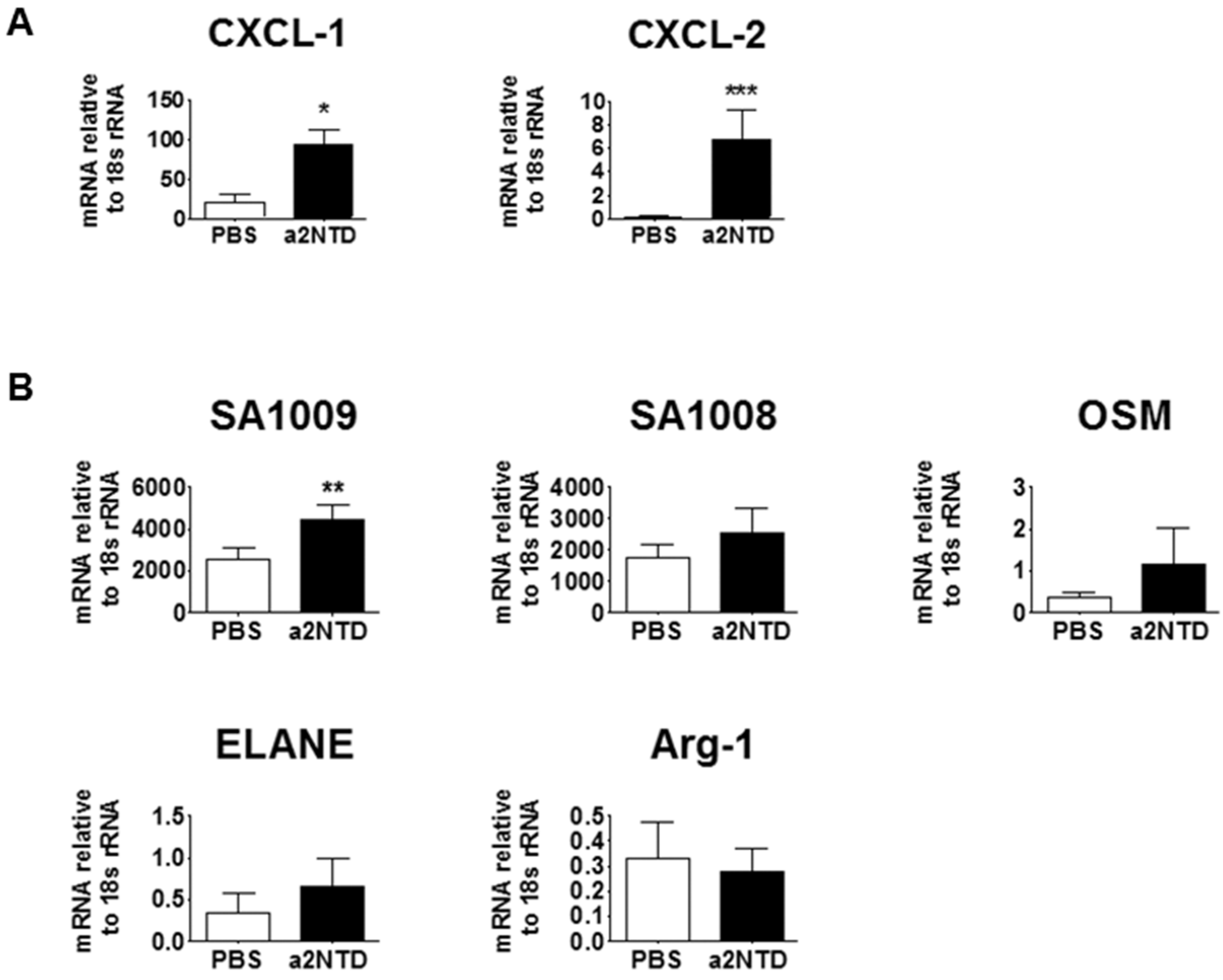

Supplementary Figure S2: Effect of a2NTD on the gene expression of Chemokines and pro-tumorigenic mediators in neutrophils. Freshly isolated human neutrophils from peripheral blood were suspended in MEM media, treated with either a2NTD or PBS as control, incubated for four hours and the mRNA expression of Chemokines; CXCL-1 and CXCL-2 **A.** as well as pro-tumorigenic mediators; 2 calcium binding proteins SA1009, SA1008, oncostatin-M (OSM), neutrophil elastase (ELANE) and arginase −1 (Arg-1) **B.** in neutrophils were assessed by quantitative real time-PCR. Data were plotted as the mean mRNA expression relative to 18s rRNA ± SEM from at least 3 individual experiments.

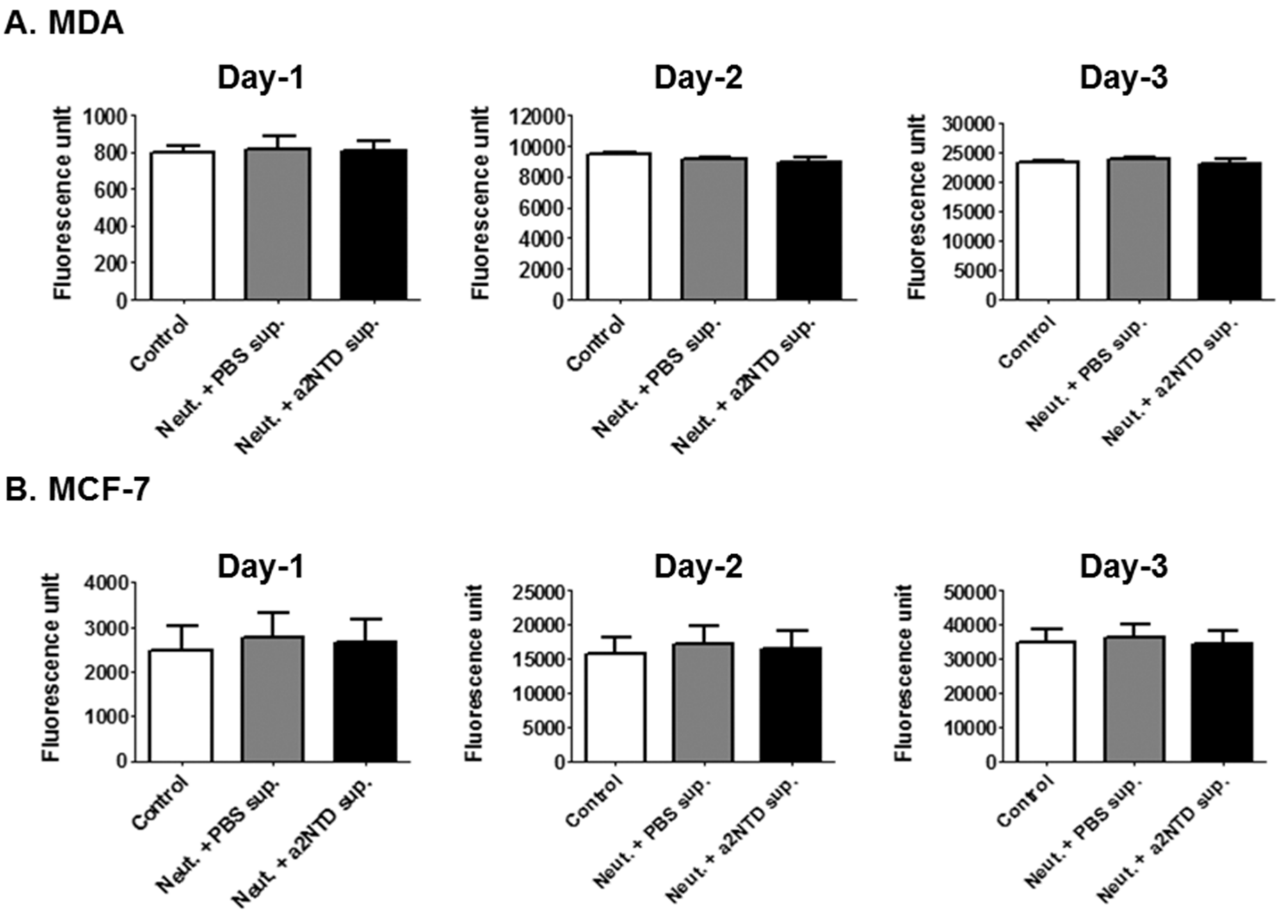

Supplementary Figure S3: a2Neuф derived products exhibit no change in breast cancer cell proliferation. *In vitro* proliferation assay was performed using Alamar blue fluorometric assay (invitrogen). 0.5 × 10^3 MDA-MB-231 **A.** or 2 × 10^3 MCF-7 **B.** were plated in a 96 well plate. Cells were cultured with either media + PBS (control), neutrophil supernatant (Neut. + PBS sup.) or a2NTD treated neutrophil supernatant (Neut. + a2NTD sup). Extent of proliferation was measured fluorometricaly after day1, 2 and 3 incubation using ELISA plate reader. Data were collected from 3 independent experiments each was done in triplicate. Results are reported as the mean fluorescence unit ± SEM.
